# Aging and CMV discordance are associated with increased immune diversity between monozygotic twins

**DOI:** 10.1186/s12979-021-00216-1

**Published:** 2021-01-18

**Authors:** Zheng Yan, Holden T. Maecker, Petter Brodin, Unni C. Nygaard, Shu Chen Lyu, Mark M. Davis, Kari C. Nadeau, Sandra Andorf

**Affiliations:** 1 Sean N. Parker Center for Allergy and Asthma Research, Stanford University School of Medicine, Stanford, CA USA; 2 Institute for Immunity, Transplantation and Infection, Stanford University School of Medicine, Stanford, CA USA; 3 Science for Life Laboratory, Department of Women’s and Children’s Health, Karolinska Institutet, Stockholm, Sweden; 4 Division of Infection Control and Environmental Health, Norwegian Institute of Public Health, Oslo, Norway; 5 Department of Pediatrics, University of Cincinnati College of Medicine, Cincinnati, OH USA; 6 Divisions of Biomedical Informatics and Allergy & Immunology, Cincinnati Children’s Hospital Medical Center, Cincinnati, OH USA

**Keywords:** Mass cytometry (CyTOF), Monozygotic twins, Aging, Cytomegalovirus, Human immunology

## Abstract

**Background:**

Broadly, much of variance in immune system phenotype has been linked to the influence of non-heritable factors rather than genetics. In particular, two non-heritable factors: aging and human cytolomegavirus (CMV) infection, have been known to account for significant inter-individual immune variance. However, many specific relationships between them and immune composition remain unclear, especially between individuals over narrower age ranges. Further exploration of these relationships may be useful for informing personalized intervention development.

**Results:**

To address this need, we evaluated 41 different cell type frequencies by mass cytometry and identified their relationships with aging and CMV seropositivity. Analyses were done using 60 healthy individuals, including 23 monozygotic twin pairs, categorized into young (12–31 years) and middle-aged (42–59 years). Aging and CMV discordance were associated with increased immune diversity between monozygotic twins overall, and particularly strongly in various T cell populations. Notably, we identified 17 and 11 cell subset frequencies as relatively influenced and uninfluenced by non-heritable factors, respectively, with results that largely matched those from studies on older-aged cohorts. Next, CD4+ T cell frequency was shown to diverge with age in twins, but with lower slope than in demographically similar non-twins, suggesting that much inter-individual variance in this cell type can be attributed to interactions between genetic and environmental factors. Several cell frequencies previously associated with memory inflation, such as CD27- CD8+ T cells and CD161+ CD4+ T cells, were positively correlated with CMV seropositivity, supporting findings that CMV infection may incur rapid aging of the immune system.

**Conclusions:**

Our study confirms previous findings that aging, even within a relatively small age range and by mid-adulthood, and CMV seropositivity, both contribute significantly to inter-individual immune diversity. Notably, we identify several key immune cell subsets that vary considerably with aging, as well as others associated with memory inflation which correlate with CMV seropositivity.

**Supplementary Information:**

The online version contains supplementary material available at 10.1186/s12979-021-00216-1.

## Introduction

The human immune system has been well known to vary considerably among individuals. Even seemingly minor differences between individuals in immune phenotype may be significant in determining relative susceptibility to both pathogenic and autoimmune diseases, as well as responsiveness to less robust drugs and vaccines [1–4]. Deeper insights into the causes of immune variance are essential for producing better therapeutics for immunity-mediated disorders, especially the ones that vary in prevalence or phenotype [5].

In order to improve understanding of immune variation, many studies have shown that, while much of immune diversity can be attributed to both heritable and non-heritable factors, non-heritable factors were often dominant in their influence [4, 6, 7]. Monozygotic (MZ) and dizygotic twins are often utilized in such studies [8–11]. One twin-study conducted by our group [12] explored the influence of aging and human cytomegalovirus (CMV) infection on immune phenotype, and found that they both account for an especially high proportion of overall immune variance. These results were supported by both previous and more recent literature [6, 13–16]. However, the extent to which non-heritable factors impact even large categories of immune cells are still under significant debate. For instance, one 2017 study on 497 female twins (aged 41–77 years) reported that inter-individual variance in adaptive immune cell diversity and activity (e.g. CD4+, CD8+ T cells) is more genetically dependent than innate immune cells (e.g. monocytes, Natural Killer T (NKT) cells) [17]. Contrastingly, another 2018 study on 1000 unrelated Western Europeans [18] found the opposite — that adaptive cells phenotypes are actually less genetically dependent than innate cell phenotypes. Thus, more information is needed about the relative contributions of genetic and non-heritable factors to the inter-individual variance for specific cell types. Such data would give broad guidance to future studies seeking to identify therapeutic targets, especially in conjunction with results from previous studies identifying immune cell frequency markers indicative of disease risk [19].

In this study, we explore the immune correlates of two major non-heritable factors: aging and human CMV seropositivity. Aging is well known to have a broad impact on immune system parameters, both through the inherent process of human development and through accumulated exposure to environmental factors [6, 12, 15, 20–22]. However, much of the literature regarding immune aging, including that published by our own group, concerns differences between young and much older (often 60 years or older) individuals [12, 19, 23], as they begin to exhibit physiological symptoms of immunosenescence [24, 25]. As a result, immune composition changes that accompany mid-adulthood (ages 40–60) are not well characterized. Studying the effects of aging on immune diversity in such middle-aged individuals would evaluate the extent to which immune aging trends previously identified in senescent individuals can already be detected by mid-adulthood, and potentially uncover new trends unidentified by previous studies. Notably, one recent study, in defining a metric of immune aging (IMM-AGE) based on longitudinal data, determined 33 cell subsets that were significantly different between young (ages 20–31) and old (aged 60–96) individuals [19]. Within these 33 cell subsets, 11 continued longitudinal dynamics within the old individuals, while 11 exhibited no significant longitudinal change with aging within either young or old individuals. These results support that significant cell compositional changes likely do occur during mid-adulthood.

Human CMV is a prevalent latent virus that has been found to shape significant portions of the immune system, altering the frequency, phenotype, and potential function of important immune cell groups [26–32]. The underlying mechanisms behind these relationships are not well studied, but suspected to be a result of memory inflation pathways [27, 33, 34]. Notably, the post-infection expansion of CMV-specific memory T cell populations has been hypothesized to have several detrimental effects, including accelerated immune aging and decreased T cell repertoire diversity, in even immunocompetent individuals [16]. These effects have been studied in mice, with results indicating that high CMV doses can impair heterologous anti-viral immunity over time [35, 36]. In another study, CMV seropositivity was significantly correlated with PCA-derived metrics characteristic of immune aging, later used as a basis for positively predicting all-cause human mortality [19]. Contrastingly, other studies have found that CMV may have positive effects to immunocompetent hosts as well, through conferring additional protection from this beneficial stimulation of the immune system [37–39]. Notably, one recent study found higher residual protection rates 1.5 years after influenza vaccination in CMV seropositive individuals [40]. Thus, a more thorough understanding of how CMV affects various cell types may lead to important insights for the risks and possibly benefits of latent viruses, informing better prognostic tools [41].

To address the question of immune system diversity based on aging and CMV, we followed up on these previous studies by further studying immune cells using mass cytometry (CyTOF) and comparing middle-aged twins (between 42 and 59 years) with younger twins (up to 31 years). We chose to investigate the effects of aging on immunity within a relatively small age-range, in order to address the aforementioned gap in knowledge. We also investigated the impact of CMV infection, a suggested accelerator of immune aging, on immune cell frequencies in this healthy MZ twin population.

## Materials and methods

### Study cohort

Data from 60 participants (23 MZ twin pairs and 14 singlets) were included in this analysis. Participants were comprised of a subset of the cohort published in Brodin et al. [12]. While we obtained the data directly from the authors, they are also publicly available through ImmPort (http://www.immport.org, IDs: SDY514, SDY515, SDY519) [42–44]. Smoking is known to have significant effects on immune composition [18]. To avoid confounding, smokers identified by the questionnaire were excluded from the participants. Individual participants were between 11.5 and 59 years old, with a median age of 28.6 (Table 1). While age was utilized as a continuous variable for fitting linear models, in the twin correlation-related analysis, we binarized age of the twins into younger (*n* = 14 pairs, 12.5–30.7 years, median of 23.6) and middle-aged (*n* = 9 pairs, 42.1–59.0 years, median of 48.6). Of the younger MZ twin pairs, 3 were CMV concordant negative, 9 were CMV concordant positive and 2 were discordant for CMV. Of the middle-aged MZ twin pairs, 5 were CMV concordant negative, 1 was CMV concordant positive and 3 were discordant for CMV.

**Table 1:** Study Cohort Demographics

	Total	Young twins	Middle-aged twins	Young singlets	Middle-aged singlets
Total participants (MZ twin pairs)	60 (23)	28 (14)	18 (9)	5	9
Female [n, %]	39 (65%)	19 (68%)	12 (67%)	3 (60%)	5 (65%)
Age (years) [median, IQR, range]	28.6, 27.0 (11.5–59.0)	23.6, 9.3 (12.5–30.7)	48.6, 10.7 (42.1–59.0)	15.8, 6.3 (11.5–22.5)	53.9, 4.0 (43.9–56.1)
Caucasian [n, %]*	47 (78%)	23 (82%)	11 (61%)	4 (80%)	9 (100%)
Asian [n, %]	10 (17%)	7 (25%)	3 (17%)	0 (0%)	0 (0%)
Black or African American [n, %]	5 (8%)	0 (0%)	4 (22%)	1 (20%)	0 (0%)
Other or declined to answer [n, %]	8 (13%)	4 (14%)	3 (17%)	1 (20%)	0 (0%)
CMV + [n, %]	32 (53%)	20 (71%)	5 (28%)	4 (80%)	6 (67%)
CMV +/+, n twin pairs (% of MZ pairs)	10 (43%)	9 (64%)	1 (11%)		
CMV −/−, n twin pairs (% of MZ pairs)	8 (35%)	3 (21%)	5 (56%)		
CMV +/−, n twin pairs (% of MZ pairs)	5 (22%)	2 (14%)	3 (33%)		

*Participants could provide several races, so that the numbers add up to more than 100%

### Immunological assays

All immunological assays were performed by the Human Immune Monitoring Center (HIMC) at Stanford University. Details are reported in Brodin et al. [12]. In brief, CMV serology was determined using a commercially available ELISA kit (CMV IgG, Gold Standard Diagnostics) as per manufacturer’s instructions. PBMCs were analyzed using mass cytometry (CyTOF, Fluidigm) with the antibody panel shown in Supplementary Table 1. From FCS3.0 files, cell subset frequencies were determined by manual gating (Supplementary Fig. 1 Supplementary Table 2) using FlowJo v9.3 (TreeStar) as also reported in [12]. In our analyses, we represent these cell subset frequencies as percentages with respect to their parent population.

### Assessing the effects of non-heritable factors on immune phenotype heritability

To determine the impacts of age on the variance of immune phenotypes, the twin-twin Spearman’s rank correlation coefficients for the frequencies of each cell type, expressed as percentages of parent populations, were compared between groups of young (*n* = 14 pairs) and middle-aged twins (*n* = 9 pairs). Similar comparisons were made between CMV discordant (−/+, *n* = 5 pairs) and CMV concordant negative (−/−, *n* = 8 pairs) twins to assess the impacts of CMV. For the correlations within the age groups and CMV concordant negative pairs, the calculation was repeated 25 times with a random break up of each twin pair into the two groups to calculate the correlations. The medians of these correlations were reported. Correlation *P* values were obtained using Spearman’s rho statistic (*cor.test* function in R). For comparison to an older group of twins, we list the Spearman’s rank correlations from the group of ≥60 year-old twins (median: 72 years; *n* = 16) from Fig. 4A in Brodin et al. [12].

Although studying Spearman’s rank correlations allows for the comparison between age groups, it does not allow the tracking of phenotypes over aging. Thus, to get a more in-depth analysis of the role of aging, we quantified how immunologically “close” each twin pair was using Euclidian distance between cell frequencies, similar to previous approaches at approximating immunological distance [45]. For comparison, this metric was also calculated between demographically similar, non-twin pairs of individuals sampled from the cohort. These non-twin pairs were generated by pairing each individual with a single random sample from the set of participants who shared their twin’s CMV status (but was not their twin) and was within +/− 5 years of age. Since each twin could be matched to between 2 and 15 (median 6) demographically similar participants outside of the twin pair, we conducted randomized non-twin pairing and Euclidean distance calculation 6 times, with medians used for the analyses and plotting. We investigated the distance between pairs in terms of all cell types to examine broad trends from aging across the immune system, as well as for CD4+ and CD8+ T cells to analyze the effects of aging on these major components of immunity.

In order to further determine the effects of CMV discordance on immune diversity, we compared the Euclidean distances across all cell type frequencies between CMV concordant positive, concordant negative, and discordant twins, as well as corresponding demographically similar non-twin pairs. Significant differences in distances were determined through the Wilcoxon rank-sum test.

*P* values were adjusted for multiple hypothesis testing using the Benjamini and Hochberg approach to control the false discovery rate (FDR) where required [46]. All statistical analyses were performed using R software (version 3.4.1).

### Determination of significant relationships between CMV and cell frequencies

To quantify directional impacts between CMV infection status and cell frequencies, a mixed model (*lmer* from package lme4 in R) was used to fit each cell type frequency. For all individuals (including singlets), age, CMV status, and sex, were used as fixed variables, while twin pair origination was used as a random variable. A chi-square test for goodness of fit was used to determine significant differences. *P* values were adjusted for multiple comparisons using the approach by Benjamini and Hochberg.

## Results

### Twin study population and assay data

Demographic data can be found in Table 1. All participants involved in the study were reported as healthy, without any symptoms of disease. A total of 64 cell types were analyzed. To avoid redundancy, a Spearman’s rank correlation filter was applied to the different cell type frequencies. In this step, of pairs of cell types with an absolute Spearman’s rank correlation of ≥0.9 between the frequencies, the cell type with the greater absolute Spearman’s rank correlation to all other cell types was chosen to be excluded. After the filter was applied, there were 41 cell types remaining (Supplementary Table 2).

### Genetically identical twins immunologically diverge from young to middle-aged

**Fig. 1:**
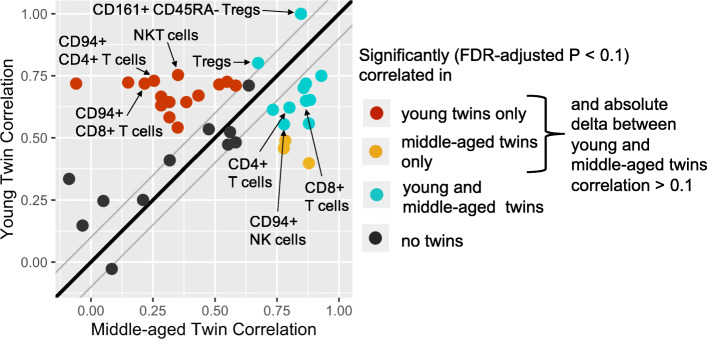
Spearman’s rank correlation (medians of random repeats; see methods) of cell frequencies for young (n = 14 pairs) vs. middle-aged (n = 9 pairs) twins. Each dot represents one cell type, colored by the significance of that correlation in the younger and middle-aged twin pairs. Black dots denote if no significant correlation was found in either young or middle-aged twins. The thick black line represents values at which the young and middle-aged twins have the exact same Spearman’s rank correlation. The upper and lower thin gray lines represent boundaries 0.1 above and below the thick black line

For each cell type, the median of the repeated calculations of the twin-twin rank correlation of the frequencies within both age groups is shown in Fig. 1 (Table 2 for corresponding values of significant [FDR-adjusted *P* < 0.1] correlations). Notably, out of the 41 cell frequencies, 17 were found to be significantly correlated only between younger twins (red dots in Fig. 1), in contrast to 3 significantly correlated only in middle-aged twins (yellow dots in Fig. 1). In particular, CD4+ and CD8+ T cells with CD94 expression both had much stronger correlations between young than between middle-aged twins, with coefficients of 0.73 and 0.72 in young twins, but were only weakly correlated with coefficients of 0.25 and 0.22 in middle-aged twins, respectively (Table 2). Other cell types only significantly correlated in young twins included NKT cells, several subsets of B cells, and CD27- and CD28- CD8+ T cells. Eleven cell types were significantly correlated in both young and middle-aged twins (teal dots in Fig. 1). These include CD4+ T cells, CD8+ T cells, CD94+ NK cells, and regulatory T cells (Tregs). Additionally reported in Table 2 are the twin-twin Spearman’s rank correlations for the group of older MZ twin pairs (≥60 years) reported in Brodin et al. [12].

**Table 2:** Cell Frequencies Significantly Correlated Between Younger and/or Middle-aged Twins

Cell type	Young twins Spearman’s rank correlation*	Middle-aged twins Spearman’s rank correlation	Young twins FDR-adjusted P	Middle-aged twins FDR-adjusted P	Older twins Spearman’s rank correlation from Brodin et al. [12]
IgD+ CD27- B cells	**0.65**	**0.88**	0.039	0.020	0.68
IgD- CD27+ B cells	**0.70**	**0.86**	0.020	0.020	0.6
IgD- CD27- B cells	**0.72**	**0.87**	0.020	0.020	0.56
CD16+ Monocytes	**0.56**	**0.88**	0.082	0.020	NA
CD4+ T cells	**0.62**	**0.80**	0.046	0.039	0.76
CD28+ CD4+ T cells	**0.75**	**0.93**	0.020	0.012	NA
CD8+ T cells	**0.65**	**0.87**	0.039	0.020	0.68
Effector Memory CD8+ T cells	**0.61**	**0.73**	0.053	0.071	0.9
CD94+ NK cells	**0.55**	**0.78**	0.083	0.039	NA
CD161+ CD45RA- Tregs	**1.00**	**0.85**	< 0.001	0.028	0.97
Tregs	**0.80**	**0.67**	0.015	0.095	0.24
B cells	**0.67**	0.43	0.038	0.34	0.25
IgD+ CD27+ B cells	**0.53**	0.47	0.095	0.27	0.51
Naïve B cells	**0.67**	0.28	0.039	0.52	0.85
Transitional B cells	**0.73**	0.55	0.020	0.19	0.28
Central Memory CD4+ T cells	**0.64**	0.38	0.041	0.40	0.55
CD94+ CD4+ T cells	**0.73**	0.25	0.020	0.56	NA
CD27+ CD4+ T cells	**0.71**	0.64	0.020	0.12	NA
CD161+ CD4+ T cells	**0.72**	0.52	0.020	0.23	NA
CD161- CD8+ T cells	**0.64**	0.32	0.040	0.47	NA
CD27- CD8+ T cells	**0.58**	0.32	0.071	0.47	NA
CD28- CD8+ T cells	**0.63**	0.28	0.045	0.52	NA
CD94+ CD8+ T cells	**0.72**	0.22	0.020	0.63	NA
CD85j + CD8+ T cells	**0.54**	0.35	0.095	0.45	NA
Effector CD8+ T cells	**0.72**	0.15	0.020	0.74	0.28
CD161- CD45RA+ Tregs	**0.72**	−0.06	0.020	0.15	0.66
CD161+ NK cells	**0.71**	0.58	0.020	0.17	NA
NKT cells	**0.75**	0.35	0.020	0.45	0.7
Lymphocytes	0.49	**0.78**	0.14	0.044	0.8
CD85j- CD4+ T cells	0.40	**0.88**	0.23	0.020	NA
Effector CD4+ T cells	0.46	**0.78**	0.19	0.039	0.7

* Medians of the repeated calculations of Spearman’s rank correlation coefficients are reported (see methods)

Statistically significant results (FDR-adjusted P < 0.1) are bolded

**Fig. 2:**
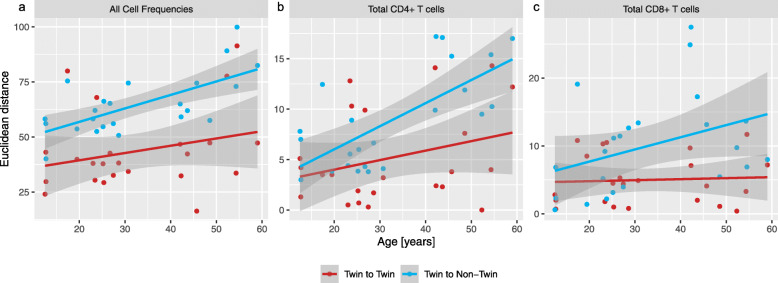
Euclidean distances between twins and between demographically similar non-twins (matched CMV status to twin and within +/− 5 years of age) over age. Each dot represents a pairwise distance for the twins or the median of the pairwise distance of repeated random matching of demographically similar non-twins, over (**a**) all cell subset frequencies, (**b**) CD4+ T cells, or (**c**) CD8+ T cells. 95% confidence intervals are shown by the shaded areas around the lines of best fit

The Euclidean distance across all cell frequencies was examined to show immune system trends with aging as a whole (Fig. 2a). Both twin-twin and non-twin immunological distance across all 41 cell frequencies correlated positively with age at slopes of 0.33 and 0.61, respectively. Interestingly, it was observed that CD4+ T cells diversified at a notably higher rate between non-twins at a slope of 0.23 compared to between MZ twins with a slope of 0.09 (Fig. 2b). In contrast, CD8+ T cells showed a median slope of 0.17 for distance between non-twins but had a comparatively constant distance (slope of 0.01) across our studied age range between twins (Fig. 2c).

### Human CMV infections contribute significantly to T cell diversification between twins

**Fig. 3:**
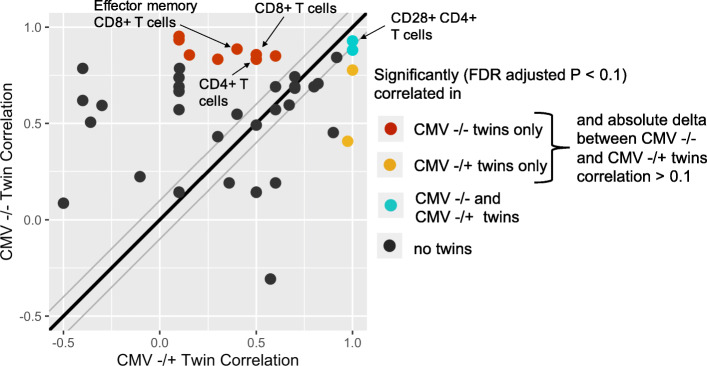
Spearman’s rank correlation plot of cell frequencies for CMV concordant negative (n = 8, medians of random repeats reported; see methods) vs. CMV discordant (n = 5) twins. Each dot represents one cell type. The thick black line represents values at which both CMV −/+ and −/− twins have the exact same Spearman’s rank correlation. The upper and lower thin gray lines represent boundaries 0.1 above and below the thick black line

It is well known that microbial exposure plays an important role in shaping the composition and function of the immune system [5]. Human CMV, a prevalent latent virus, is one such microbe known to have broad and lasting effects on immune phenotype [12, 27, 28, 47]. In our twin cohort, 5 pairs of CMV discordant (+/−) twins were identified and their twin-twin Spearman’s rank correlations across all cell frequencies were compared with all 8 pairs of CMV concordant negative (−/−) twins (Fig. 3, Table 3). Several cell frequencies showed higher correlations in CMV concordant negative twins than CMV discordant twins, supporting previous findings that CMV has a broad impact on the immune system [12]. Various T cell subsets were especially affected, including CD4+ T cells, effector memory CD8+ T cells, and CD8+ T cells, each having strong, significant correlations (FDR-adjusted *P* < 0.1) in only CMV negative concordant twins. Two cell populations, namely IgD- CD27- B cells and CD28+ CD4+ T cells, were significantly correlated between twins in both CMV negative concordant and discordant groups, suggesting these populations are especially stable across persistent viral stimulation.

**Table 3:** Cell Frequencies Significantly Correlated Between CMV−/− (n = 8 pairs) and/or CMV−/+ (n = 5 pairs) Twins

Cell type	CMV −/− twins Spearman’s rank correlation*	CMV −/+ twins Spearman’s rank correlation	CMV −/− twins FDR-adjusted P	CMV −/+ twins FDR-adjusted P
IgD- CD27- B cells	**0.88**	**1.00**	0.077	0.091
CD28+ CD4+ T cells	**0.93**	**1.00**	0.061	0.091
IgD+ CD27- B cells	**0.95**	0.10	0.047	0.95
IgD- CD27+ B cells	**0.83**	0.30	0.085	0.81
Transitional B cells	**0.85**	0.60	0.077	0.56
CD4+ T cells	**0.83**	0.50	0.091	0.66
CD85j- CD4+ T cells	**0.93**	0.10	0.047	0.95
CD8+ T cells	**0.86**	0.50	0.085	0.66
Effector memory CD8+ T cells	**0.89**	0.40	0.069	0.69
CD161+ CD45RA- Tregs	**0.84**	0.92	0.078	0.12
NKT cells	**0.86**	0.15	0.077	0.92
CD94+ NK cells	0.41	**0.97**	0.55	0.077
Tregs	0.78	**1.00**	0.12	0.091

* For the CMV concordant negative twins, medians of the repeated calclations of Spearman’s rank correlations are reported (see methods)

Statistically significant results (FDR-adjusted P < 0.1) are bolded

**Fig. 4:**
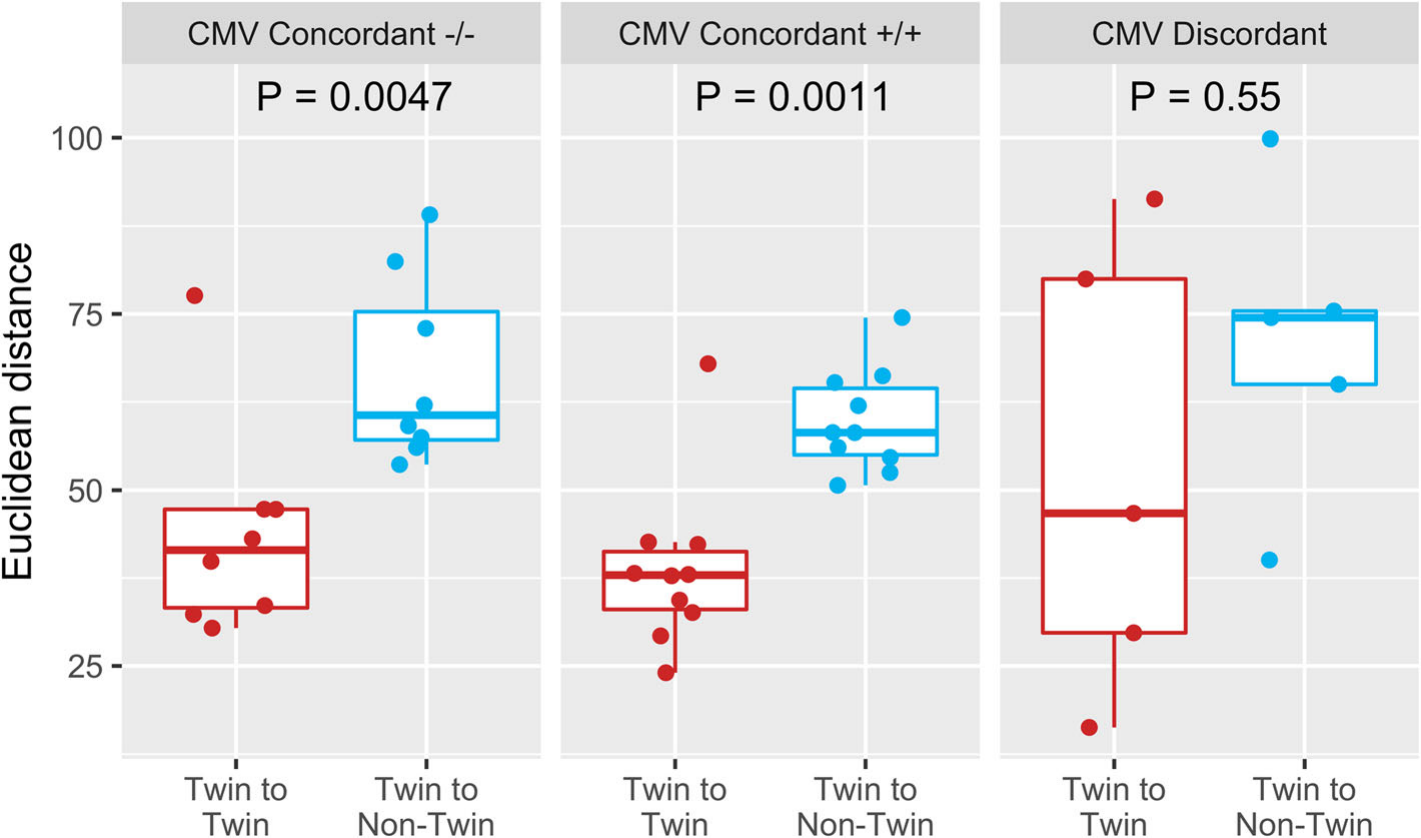
Euclidean distance for all cell subset frequencies between twin and demographically similar non-twin pairs (median distance for 6 repeated random matching of demographically similar individuals), stratified by pairwise CMV seropositivity. Each dot represents the immunological distance, taking into account all cell frequencies, between a twin or non-twin pairing. P values by Wilcoxon rank sum tests

To further examine the impacts of CMV status on immune cell diversity, both twin-to-twin and demographically similar non-twin Euclidean distances were calculated across all cell types and categorized by individual CMV seropositivity (Fig. 4). There was a significant difference between twin-twin and twin-non-twin distance for all CMV concordant twins (*P* < 0.01), but not between CMV discordant twins (*P* = 0.55). This analysis supports previous findings that CMV seropositivity, although often symptomless, additionally shapes the immune system in many ways.

### CMV infection increases anti-inflammatory and viral-specific memory T cell frequencies

**Fig. 5:**
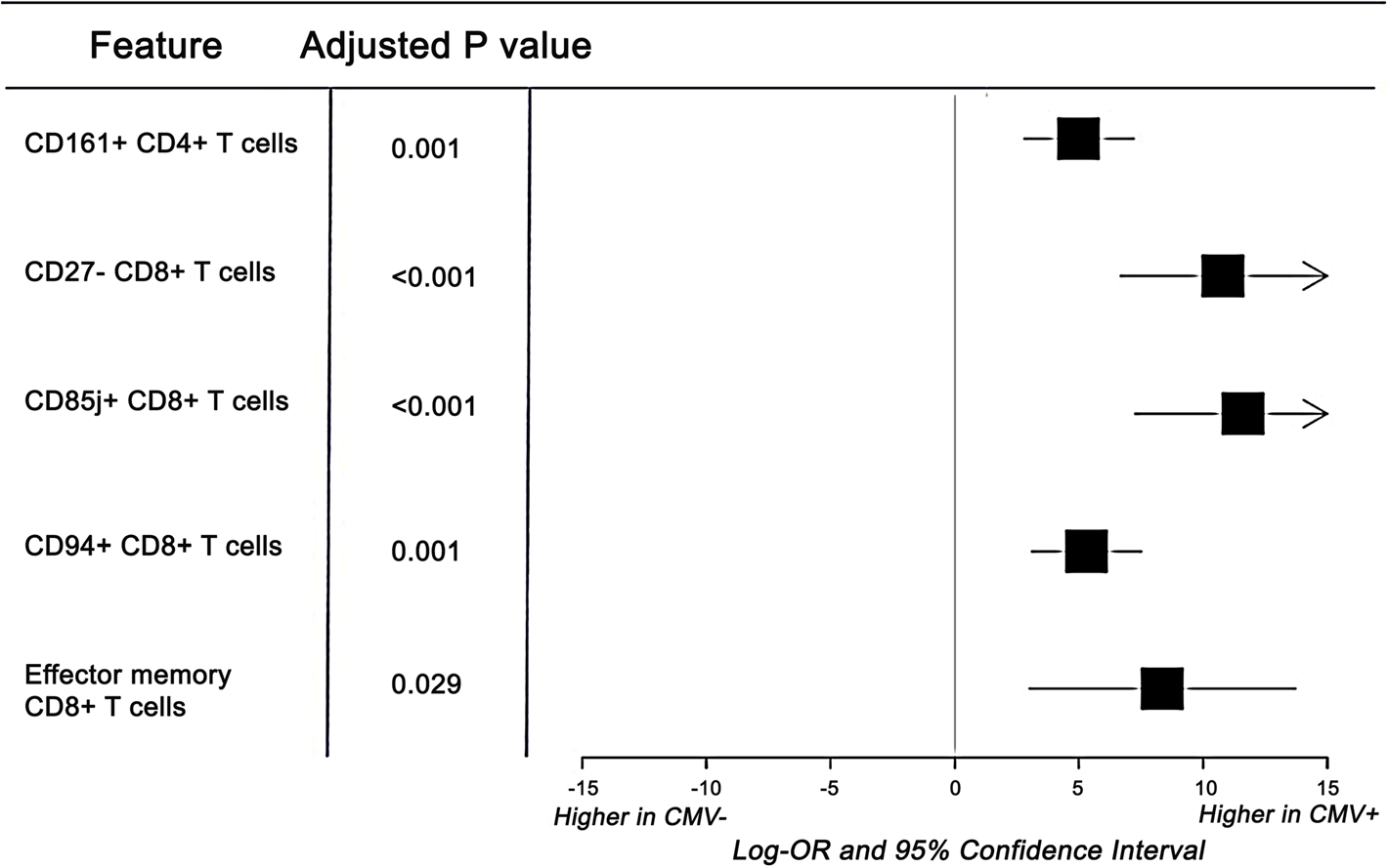
Cell frequencies significantly correlated with CMV positivity in our cohort. Each row in the forest plot represents a cell subset for which the frequency was found to be significantly correlated (FDR-adjusted *P* value < 0.05) with CMV positivity within the cohort (after adjustment for age, sex and twin pair (or singlet) origin). The log odds ratio using the features of interest to predict CMV positivity are also shown as measurements of effect size, along with corresponding confidence intervals

To quantify directional effects, we also looked for significant correlations (FDR-adjusted *P* < 0.05) between cell frequencies and both aging and CMV. CD94+ CD8+ T cells and CD85j + CD8+ T cells, both cell types associated with inhibition of cytotoxicity [26, 27], were found to have significantly higher frequencies in CMV positive individuals (Fig. 5). CD161+ CD4+ T cells, effector memory CD8+ T cells, and CD27- CD8+ T cells, indicators of long-term viral-specific memory pools [16, 29], were found to have significantly higher frequencies in CMV positive individuals as well.

## Discussion

To further understand the effects of inherited vs non-inherited influences on the immune system, we analyzed the impact of age and CMV status on the cellular composition of the immune system in a cohort of 46 MZ twins (23 pairs) in this exploratory, pilot study.

Firstly, we examined the effects of being middle-aged (median 48.6 years) vs younger (median 23.6 years) on cell frequency diversity between healthy MZ twins by Spearman’s rank correlation coefficient. A subset (17 out of 41) of the cell frequencies were observed to diverge in the middle-aged MZ twins, but not younger ones. This result supports that aging has a large impact on the variability of several adaptive cell types, many of which have also been found to vary with age by previously published studies [12, 45]. In a similar analysis by our group [12], we compared young MZ twins (≤ 20 years, median 13.5) with older twins (≥ 60 years, median 72), and highlighted Tregs as the most extreme example of being very strongly correlated (coefficient 0.78) in young twins and not correlated (coefficient 0.24) in the older twins. In this current study, for Treg frequencies we observed a strong correlation for the younger twins (coefficient 0.80), but just slightly weaker correlation for the middle-aged twins (coefficient 0.67) (Table 2). These results, taken together, are in line with prior studies indicating that changes in Treg frequency occur more dominantly in older adults [48–50]. In the data presented here, 11 out of the 41 cell frequencies were found to have significant correlations between twins of both studied age groups. The cell types were mostly consistent with what was reported as being dominantly controlled by heritable factors when analyzed using a structural equation modeling approach including dizygotic twins to estimate heritability [12]. All of these cell types, except Tregs, for which correlation values were reported in Brodin et al. [12] had also for the older twin population Spearman’s rank correlations of at least 0.56. Overall, these results support that, between pre-senescent individuals, immune cell composition tends to diverge alongside aging in some areas but remain stable in others. Specifically, we highlighted 17 and 11 cell subset frequencies as relatively influenced and uninfluenced by non-heritable factors, respectively. These 17 cell subsets that diverged in middle-aged MZ twins are more likely to be affected by environmental or lifestyle factors and are thus particularly interesting targets for future precision health studies, especially those cell types previously associated with risk factors. For instance, CD28- CD8+ T cell and B cell frequencies were also major components of the cell-based IMM-AGE score, whose approximation was found to be a meaningful predictor of all-cause mortality in a dataset comprised of both middle aged and older individuals [19]. As mentioned in the introduction, 11 cell subsets were identified in that study as showing a significant difference between the young and old individuals and as demonstrating continued longitudinal changes in the old individuals. Interestingly, 7 of these cell subsets were also analyzed in our work and 4 (B cells, CD161- CD45RA+ Treg, CD28- CD8+ T cells, Effector CD8+ T cells) showed a significant correlation in the younger twins but not in the middle-aged twins. This could suggest that non-heritable factor driven changes of these cell subsets start at early mid-age and continue into older adulthood. To make rigorous conclusions, future longitudinal studies spanning mid-age and older individuals should investigate the dynamics of these cell subsets.

In interpreting our results, several limitations should be kept in mind. First, we utilized previously generated data in this pilot study and the sample size is limited. As a result, many of the discussed findings are preliminary and should be tested in a larger cohort. Furthermore, younger twins often either cohabitate or have lived apart for a shorter period of time, confounding these findings due to more recently shared environmental exposures. Lastly, the participants in this study were mostly Caucasian, making the presented results strongly biased towards this ethnicity.

To calculate twin-twin cell frequency correlations within an age group, we first randomly assigned each member of every eligible twin pair into either the “twin A” or “twin B” group. However, especially given the small sample size, exactly how twins were divided into these two groups may contribute unnecessary variance. To mitigate this variance, we repeated this split of twins randomly 25 times and recorded not only the median Spearman’s rank correlation and *P* value as reported in the results, but also the interquartile range (IQR) of the correlation coefficients of the 25 repeats per cell type per age group. Across the cell types, the IQR of the correlation coefficients in the younger twins ranged from 0 to 0.14 and in the middle-aged twins from 0.012 to 0.19. This variability can be attributed to the small sample size and needs to be considered when interpreting our results.

As an additional approach to quantifying immune variance, distances were calculated both between twins and between non-twins using a similar Euclidean approach as previously done to approximate immunological closeness (Fig. 2) [45]. Importantly, non-twin pairs were generated by matching individuals to a randomly sample from the set of participants similar to their twin, as defined by age and CMV seropositivity. Interestingly, we observed that CD4+ T cells diversified over age between twins as well as non-twins; however, we found a higher rate of divergence in the non-twins, suggesting that much of inter-individual variance within this cell type may be attributed to interactions between factors regulated by environment and factors driven by genetics. Note that while our results highlight differing rates of divergence in the immune system between MZ twins and non-twins, to estimate actual heritability, the inclusion of dizygotic twins is needed, as was done in Mangino et al. [17]. Previous work in this area has shown conflicting results, which may stem from the highly heterogenous consistency of CD4+ T cell populations. For instance, while Brodin et al. [12] and Patin et al. [18] identified CD4+ T cell proportions as non-genetically determined, Mangino et al. found CD4+ T cell proportions to be more influenced by genetics [17]. In contrast to CD4+ T cells, our data for CD8+ T cells showed no increase of distance between twins over age (Fig. 2c). Prior findings showed CD8+ T cell population frequencies as being most likely more genetically determined [12, 18, 51]. Overall, genetically identical twins might have the tendency to react similarly to accumulated environmental exposure, causing their rate of immune divergence to be lower than that of non-twins but nevertheless showing an increased distance over age. Thus, our results support that gene-environment interactions play important roles in determining immune system composition over time, and specifically within T cell populations. To make rigorous conclusions in this area, both additional multi-omics data and larger sample sizes including MZ and dizygotic twins are needed, especially since we observed large variations in distances among twin pairs.

CMV, one of the most common viral infections in the world, and recently a suspect in causing significant immune debilitations [16, 30, 52], was another focus of our study. Our results suggest that CMV discordance increases immune diversity between twins (Fig. 3), showing that a large set of immune cells are impacted by CMV, as previously publications have found [26, 28, 32]. Twin-twin averaged immunological distances were significantly lower than non-twin distances for both CMV concordant positive and CMV concordant negative twins, but not between CMV discordant twins (Fig. 4). However, these limited results should only be interpreted as possible trends because of the low sample size (*n* = 5) of CMV discordant twin pairs in our cohort.

When comparing the results of the correlation analyses based on age and CMV, NKT cells and transitional B cells were found to be significantly correlated between young twins but not in middle-aged twins and were also significantly correlated in CMV negative concordant but not CMV discordant twins. Recent publications investigating the immunogenic effects of CMV have demonstrated similar results, suggesting that CMV may accelerate aging in many areas of immune phenotype [45]. Due to sample size limitations, we were unable to test this hypothesis more strongly through directly comparing cell frequency correlations between young CMV discordant twins and between older CMV concordant twins. A previous study suggested that the immune response to CMV may also be genetically influenced [53], underscoring the need for such studies in larger CMV-related cohorts including MZ and dizygotic twins.

Furthermore, we found several cell types to be significantly correlated with CMV seropositivity in the overall cohort (Fig. 5). To begin, one other study also examined the association between CD161+ CD4+ T cells and CMV seropositivity, finding no difference in their frequency between CMV+ and CMV- young individuals [54]. Contrastingly, we found that CMV seropositivity increases CD161+ CD4+ T cell levels in our cohort, which consists of both young and middle-aged individuals, while controlling for age. The previous study also found that CD161+ CD4+ T cells decrease in age within CMV seropositive individuals. Together, these results suggest that age and CMV seropositivity potentially both impact CD161+ CD4+ T cell levels. As this cell subset has been found to contribute to long-term persistence of virus-specific memory CD4+ T cells, future studies in this area may reveal more insights about how different conditions affect immune memory [29]. Next, our finding of significantly greater levels of CD85j + CD8+ T cells and CD94+ CD8+ T cells in CMV positive individuals than in CMV negative participants is in line with prior findings [26, 27, 55]. The precise role of CD85j and CD94, which are traditionally NK receptors, in the CMV-initiated T cell response has yet to be elucidated [56]. One study hypothesized that CD85j plays an important role in CMV-mediated T cell dysfunction – as CD85j is induced by persistent antigen exposure, inhibits effector function by blocking interferon production, and can be activated by the CMV MHC homolog UL18 [55]. Lastly, we found significantly higher levels of CD27- CD8+ T cells and effector memory CD8+ T cells in CMV positive participants, which were previously characterized as memory inflation phenotypes and have been previously observed in both mice and humans [26, 27, 57, 58]. These results support prior hypotheses that, through persistent and specific immune responses that dominate existing memory pools, CMV accelerates immune aging and leads to increased immune dysfunction [14, 16, 52, 59]. One study showed that upregulation of these cell types, alongside other late-differentiated CD4+ cells, in CMV-positive individuals were influenced by genetic factors as well [53]. In the future, further multi-omics analyses may provide more useful insights for the implications of CMV infection on an individual’s immune composition.

Lastly, while CMV is the most studied virus in relation to immunosenescence, studies suggest that more viruses as well as remnants of ancient retroviral infections in our genome potentially affect immune aging, as discussed in a recent review [60]. Notably, it is estimated that adults are infected by 5–10 persistent/chronic viruses, many of which were also associated with immunosenescence [52, 61]. Future studies exploring factors associated with immune aging should therefore include these factors in addition to CMV.

## Conclusions

Our exploratory study finds that aging, even within our relatively small age range, and CMV seropositivity are major sources of immune variance. Notably, we identified 17 cell frequencies which significantly correlate between younger twins, but not middle-aged ones – indicating that these subsets are most susceptible to influence by environmental factors. These cell frequencies were largely also identified in similar studies on young vs. older individuals, suggesting that for this subset of cell types, this effect of increased immune diversity between monozygotic twins is already notable within a relatively small age range and by mid-adulthood. In the future, the specific origins and implications of variance in these subsets will likely be beneficial to study, such as for developing more personalized immunological therapies and for studying immunotoxic effects of environmental factors. We also highlight several cell subsets, many previously associated with the phenomenon of memory inflation, to be correlated with CMV seropositivity. This evidence supports previous findings that CMV may reduce T cell diversity, potentially leading to decreased adaptive immune function.

## Supplementary Information

**Additional file 1: Supplementary Fig. 1.** Manual gating schema using the FlowJo v9.3 software (TreeStar, Inc) for one representative FCS3.0-file generated by Mass Cytometry (CyTOF). **Supplementary Table 1.** CyTOF panel used for data acquisition at the Human Immune Monitoring Center (HIMC) at Stanford University. All antibodies were conjugated in house. **Supplementary Table 2.** Manual gating strategy for the 41 cell types remaining after filtering for our analysis.

## Data Availability

The dataset supporting the conclusions of this article is available in ImmPort, [http://www.immport.org, IDs: SDY514, SDY515, SDY519].

## Abbreviations

CMV: cytolomegavirus
FDR: false discovery rate
IQR: interquartile range
MZ: monozygotic
NKT: Natural Killer T
Tregs: regulatory T cells
