# Supplementary material for: Aging and CMV discordance are associated with increased immune diversity between monozygotic twins
Source: Immun Ageing. 2021 Jan 18;18:5. doi: 10.1186/s12979-021-00216-1 (PMC7812659; doi:10.1186/s12979-021-00216-1)
Supplement: Supplementary file 1 — Additional file 1: Supplementary Fig. 1. Manual gating schema using the FlowJo v9.3 software (TreeStar, Inc) for one representative FCS3.0-file generated by Mass Cytometry (CyTOF). Supplementary Table 1. CyTOF panel used for data acquisition at the Human Immune Monitoring Center (HIMC) at Stanford University. All antibodies were conjugated in house. Supplementary Table 2. Manual gating strategy for the 41 cell types remaining after filtering for our analysis. [file 12979_2021_216_MOESM1_ESM.docx]

# Supplementary Figures and Tables

#

**Supplementary Figure 1.** Manual gating schema using the FlowJo v9.3 software (TreeStar, Inc) for one representative FCS3.0-file generated by Mass Cytometry (CyTOF).

**Supplementary Table 1.** CyTOF panel used for data acquisition at the Human Immune Monitoring Center (HIMC) at Stanford University. All antibodies were conjugated in house.

| **Metal label** | **Specificity** | **Clone** |
| --- | --- | --- |
| 115In | live/dead |  |
| 142Nd | CD19 | SJ25C1, Southern BioTech |
| 143Nd | CD4 | SK3, BioLegend |
| 144Nd | CD8 | SK1, BioLegend |
| 146Nd | IgD | IA6-2, BioLegend |
| 147Sm | CD85j | 292319, R&D Systems |
| 149Sm | CD16 | 3G8, BioLegend |
| 150Nd | CD3 | UCHT1, BD |
| 151Eu | CD38 | HB-7, BD |
| 152Sm | CD27 | L128, BD |
| 154Sm | CD14 | M5E2, BioLegend |
| 156Gd | CD94 | HP-3D9, BD |
| 160Gd | CCR7 | 150503, R&D Systems |
| 162Dy | CD45RA | HI100, BioLegend |
| 164Dy | CD20 | 2H7, BioLegend |
| 165Ho | CD127 | A019D5, BioLegend |
| 166Er | CD33 | P67.8, BD |
| 167Er | CD28 | L293, BD |
| 168Er | CD24 | ML5, BioLegend |
| 170Er | CD161 | DX12, BD |
| 171Yb | TCRgd | B1, BioLegend |
| 174Yb | CD56 | NCAM16.2, BD |
| 175Lu | HLA-DR | G46-6, BD |
| 176Yb | CD25 | M-A251, BD |

**Supplementary Table 2.** Manual gating strategy for the 41 cell types remaining after filtering for our analysis.

| B cells | |
| --- | --- |
| B cells | CD14-/CD33-/CD3-/CD19+/CD20+ |
| IgD+CD27+ B cells | CD14-/CD33-/CD3-/CD19+/CD20+/IgD+/CD27+ |
| IgD+CD27- B cells | CD14-/CD33-/CD3-/CD19+/CD20+/IgD+/CD27- |
| IgD-CD27+ B cells | CD14-/CD33-/CD3-/CD19+/CD20+/IgD-/CD27+ |
| IgD-CD27- B cells | CD14-/CD33-/CD3-/CD19+/CD20+/IgD-/CD27- |
| naïve B cells | CD14-/CD33-/CD3-/CD19+/CD20+/CD24-/CD38+ |
| plasmablasts | CD14-/CD33-/CD3-/CD20-/CD27+/CD38+ |
| transitional B cells | CD14-/CD33-/CD3-/CD19+/CD20+/CD24+/CD38+ |
|  |  |
| CD16+ monocytes | CD14+/CD33+/CD16+ |
| CD161+ NK cells | CD14-/CD33-/CD3-/CD16+/CD56+/CD161+ |
| CD94+ NK cells | CD14-/CD33-/CD3-/CD16+/CD56+/CD94+ |
| HLADR+ NK cells | CD14-/CD33-/CD3-/CD16+/CD56+/HLADR+ |
| lymphocytes | CD14-/CD33- |
| NK cells | CD14-/CD33-/CD3-/CD16+/CD56+ |
| non-T lymphocytes | CD14-/CD33-/CD3- |
|  |  |
| CD161+CD4+ T cells | |
| CD161+CD4+ T cells | CD14-/CD33-/CD3+/CD4+/CD161+ |
| CD161+CD45RA- Tregs | CD14-/CD33-/CD3+/CD4+/CD25hi/CD127low/CD161+/CD45RA- |
| CD161-CD45RA+ Tregs | CD14-/CD33-/CD3+/CD4+/CD25hi/CD127low/CD161-/CD45RA+ |
| CD161-CD8+ T cells | CD14-/CD33-/CD3+/CD8+/CD161- |
| CD27-CD8+ T cells | CD14-/CD33-/CD3+/CD8+/CD27- |
| CD28-CD8+ T cells | CD14-/CD33-/CD3+/CD8+/CD28- |
| CD4+ T cells | CD14-/CD33-/CD3+/CD4+ |
| CD4+CD27+ T cells | CD14-/CD33-/CD3+/CD4+/CD27+ |
| CD4+CD28+ T cells | CD14-/CD33-/CD3+/CD4+/CD28+ |
| CD8+ T cells | CD14-/CD33-/CD3+/CD8+ |
| CD85j+CD8+ T cells | CD14-/CD33-/CD3+/CD8+/CD85j+ |
| CD85j-CD4+ T cells | CD14-/CD33-/CD3+/CD4+/CD85j- |
| CD94+CD4+ T cells | CD14-/CD33-/CD3+/CD4+/CD94+ |
| CD94+CD8+ T cells | CD14-/CD33-/CD3+/CD8+/CD94+ |
| central memory CD4+ T cells | CD14-/CD33-/CD3+/CD4+/CCR7+/CD45RA- |
| central memory CD8+ T cells | CD14-/CD33-/CD3+/CD8+/CCR7+/CD45RA- |
| effector CD4+ T cells | CD14-/CD33-/CD3+/CD4+/CCR7-/CD45RA+ |
| effector CD8+ T cells | CD14-/CD33-/CD3+/CD8+/CCR7-/CD45RA+ |
| effector memory CD4+ T cells | CD14-/CD33-/CD3+/CD4+/CCR7-/CD45RA- |
| effector memory CD8+ T cells | CD14-/CD33-/CD3+/CD8+/CCR7-/CD45RA- |
| gamma-delta T cells | CD14-/CD33-/TCRgd+ |
| HLADR-CD38+CD4+ T cells | CD14-/CD33-/CD3+/CD4+/HLADR-/CD38+ |
| HLADR-CD38+CD8+ T cells | CD14-/CD33-/CD3+/CD8+/HLADR-/CD38+ |
| naive CD4+ T cells | CD14-/CD33-/CD3+/CD4+/CCR7+/CD45RA+ |
| NKT cells | CD14-/CD33-/CD3+/CD56+ |
| Tregs | CD14-/CD33-/CD3+/CD4+/CD25hi/CD127low |
